# Effects of non-coding RNAs and RNA-binding proteins on mitochondrial dysfunction in diabetic cardiomyopathy

**DOI:** 10.3389/fcvm.2023.1165302

**Published:** 2023-09-01

**Authors:** Koray N. Potel, Victoria A. Cornelius, Andrew Yacoub, Ali Chokr, Clare L. Donaghy, Sophia Kelaini, Magdalini Eleftheriadou, Andriana Margariti

**Affiliations:** ^1^ Wellcome-Wolfson Institute for Experimental Medicine, School of Medicine, Dentistry and Biomedical Sciences, Queen’s University Belfast, Belfast, United Kingdom; ^2^ Faculty of Medicine, University of Picardie Jules Verne, Amiens, France

**Keywords:** diabetes mellitus, diabetic cardiomyopathy, mitochondrial dysfunction, noncoding RNAs, RNA-binding proteins, stem cell modelling

## Abstract

Vascular complications are the main cause of diabetes mellitus-associated morbidity and mortality. Oxidative stress and metabolic dysfunction underly injury to the vascular endothelium and myocardium, resulting in diabetic angiopathy and cardiomyopathy. Mitochondrial dysfunction has been shown to play an important role in cardiomyopathic disruptions of key cellular functions, including energy metabolism and oxidative balance. Both non-coding RNAs and RNA-binding proteins are implicated in diabetic cardiomyopathy, however, their impact on mitochondrial dysfunction in the context of this disease is largely unknown. Elucidating the effects of non-coding RNAs and RNA-binding proteins on mitochondrial pathways in diabetic cardiomyopathy would allow further insights into the pathophysiological mechanisms underlying diabetic vascular complications and could facilitate the development of new therapeutic strategies. Stem cell-based models can facilitate the study of non-coding RNAs and RNA-binding proteins and their unique characteristics make them a promising tool to improve our understanding of mitochondrial dysfunction and vascular complications in diabetes.

## 1. Background

Diabetes mellitus (DM) and its complications are a major global health burden, affecting millions of patients worldwide (1). Amongst the various macro- and microvascular complications of DM, diabetic cardiomyopathy (DCM) is a critical cause of DM-associated morbidity and mortality. DCM is characterized by cardiac dysfunction and heart failure in the absence of hypertension, coronary artery disease or other cardiac pathologies (2, 3). This dysfunction is driven by the molecular changes seen in DM, including hyperglycemia, increased fatty acid metabolism, inflammation and fibrosis, which collectively contribute to the pathophysiology of DCM (4). In recent years, a role for mitochondrial dysfunction in DCM has emerged also.

**Figure 1:**
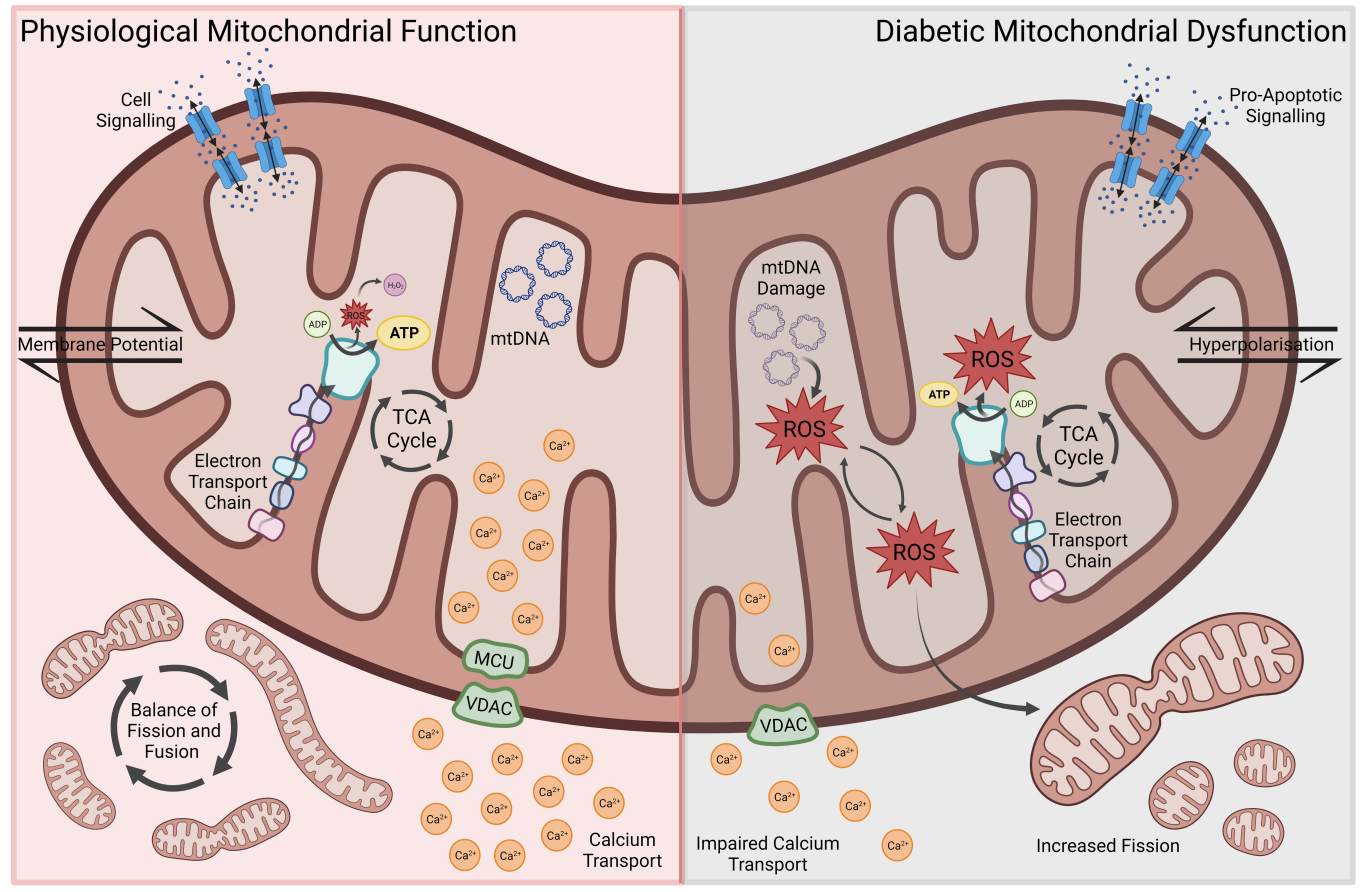
Aspects of mitochondrial dysfunction seen in diabetic cardiomyopathy, including reduced energy metabolic efficiency, ROS-associated oxidative stress and mitochondrial DNA damage, impaired Ca^2+^ transport, increased mitochondrial fission, membrane hyperpolarization and pro-apoptotic signaling. ROS, reactive oxygen species; MCU, mitochondrial calcium uniporter; VDAC, voltage-dependent anion channel.

Whilst mitochondria are regarded primarily for their central role in bioenergetic and biosynthetic pathways, within the last few decades a greater recognition for the role of mitochondria in a range of cellular processes such as regulation of ion homeostasis, cell growth, redox status, signal transduction, immunity and cell survival has been gained (5–7). Accordingly, an appreciation for the role of mitochondrial dysfunction as a major contributing factor to multiple disease states, including neurodegenerative disorders, diabetes and cardiovascular diseases, has emerged (8, 9). Modulated by genetics, lifestyle and environment, mitochondrial dysfunction is therefore now accepted to not only underlie mitochondrial diseases through the mutation of either genes encoding mitochondrial proteins or non-mitochondrial genes involved in mitochondrial biology, but also a repertoire of complex disease states. Due to the heart's high demand for adenosine triphosphate (ATP), mitochondria are found more abundantly within cardiomyocytes. A study revealed between 20% and 40% of the volume within adult cardiomyocytes to consist of mitochondria (10). It is therefore not surprising, that mitochondrial dysfunction, and the associated disruptions in energy metabolism, oxidative balance, calcium handling and apoptosis, have emerged as key drivers of cardiac dysfunction and the pathophysiological changes in DCM (Figure 1) (3, 11, 12).

In the healthy heart, about 70% of energy is derived from the oxidation of fatty acids. The source of the remaining 30% comprise glucose, amino acids and other nutrients. While the oxidation of fatty acids is not as efficient as the metabolism of glucose or other substrates, requiring more oxygen to generate the same amount of ATP, there exists a physiological balance between energy need, oxygen consumption and ATP synthesis. In the diabetic heart, however, a shift towards increased fatty acid metabolism results in an increased oxygen demand and reduced cardiac efficiency (11, 13). This metabolic shift can be already observed in the hearts of obese human subjects, manifesting as increased oxygen consumption alongside enhanced uptake and oxidation of fatty acids (14). Montaigne et al. showed, however, that contractile impairment and mitochondrial dysfunction can develop in DCM irrespective of weight. Furthermore, they found mitochondrial dysfunction to be directly and independently associated with hemoglobin A1c (HbA1c) levels—highlighting the importance of hyperglycemia and decreased glucose uptake in the development of these metabolic changes (15). In various animal models of DCM, a reduced mitochondrial oxidative capacity and efficiency can be observed. Additionally, cardiomyocytes seem to be unable to sufficiently adapt their substrate utilization in response to shifting levels of glucose and fatty acids, resulting in increased fatty acid metabolism and oxygen demand irrespective of substrate availability (16–19). The peroxisome proliferator-activated receptor ɑ (PPARɑ) plays a central role in shifting mitochondrial metabolism towards increased fatty acid oxidation (12, 20). PPARɑ is a member of the ligand-activated nuclear receptor superfamily and major gene expression regulator of enzymes involved in fatty acid β-oxidation. Increased lipid uptake induces PPARɑ which in turn upregulates enzyme expression (21). In mice with cardiac-specific overexpression of PPARɑ, a DCM-like phenotype can be observed, including characteristic metabolic shifts and cardiac dysfunction (22). Further elucidating the role of PPARɑ in the development of DCM, Buchanan et al. found increased fatty acid metabolism and the development of cardiac dysfunction to precede PPARɑ-overexpression in obese and diabetic mice, which indicates that there are co-existing mechanisms driving early metabolic and phenotypic changes (23).

Alongside the reduced mitochondrial oxidative capacity and efficiency, increased oxidative stress has been identified as a key driver of DCM pathogenesis. During mitochondrial ATP production through the respiratory chain, reactive oxygen species (ROS) are produced, such as superoxide anion (O_2_^−^). Whilst normal amounts of ROS can be converted to more stable forms including O_2_^−^ into hydrogen peroxide, overproduction of ROS creates oxidative stress resulting in damage to mitochondrial DNA via mutations, altered membrane permeability, calcium homeostasis and mitochondrial defense systems. Moreover, damage to mitochondrial DNA has been shown to increase ROS and oxidative stress creating a harmful cycle of distress (24, 25). Due to the abundance of mitochondria in cardiac tissue, mitochondrial dysfunction and ROS production are thought to contribute significantly to cardiac pathologies (26–29). In diabetic mice, increased myocardial oxygen consumption and cardiac dysfunction coincide with augmented generation of ROS as well as lipid and protein peroxidation products (30). Similarly, mitochondrial dysfunction and increased oxidative stress are seen in rats with DCM (31). This increase in oxidative stress promotes cardiac cell death (32). Insulin treatment, on the other hand, can prevent free radical damage and reverse mitochondrial dysfunction in diabetic hearts (33).

Besides their role in energy metabolism and oxidative balance, mitochondria are also involved in the intracellular handling and storage of calcium. Ca^2+^ is a central component of cardiomyocyte contraction and signaling, which is why disruptions of Ca^2+^ homeostasis secondary to mitochondrial dysfunction contribute to the pathophysiological features of DCM. In a reciprocal regulatory relationship, cytosolic Ca^2+^ mediates both ATP production and utilization, by influencing mitochondrial oxidation, enhancing aerobic respiration and modulating ATPase activity (34–36). Cytosolic and mitochondrial Ca^2+^ levels fluctuate throughout the cardiac contraction cycle. Mitochondria take up Ca^2+^ during systole and release it into the cytoplasm throughout diastole (37). Allowing the flow of Ca^2+^ from the cytoplasm into the mitochondrial matrix is the mitochondrial Ca^2+^ uniporter (MCU), which is downregulated in diabetes. The MCU plays a vital role in intracellular Ca^2+^ homeostasis and signaling. Its downregulation in diabetes leads to impaired mitochondrial Ca^2+^ uptake and was found to be associated with a decreased capacity to upregulate ATP synthesis in response to increased workload, resulting in contractile dysfunction (38). Upregulation of MCU in diabetic animal models restored normal energy metabolism and mitochondrial Ca^2+^ handling, while decreasing oxidative stress and cardiomyocyte apoptosis (39, 40). Other key ion channels found to be downregulated in diabetes include sarco-endoplasmic reticulum calcium ATPase (SERCA) and Ryanodine Receptor 2 (RYR2), which are responsible for sarcoplasmic Ca^2+^ uptake and release, respectively. Their downregulation directly impacts cytosolic and mitochondrial Ca^2+^ levels, disrupting intracellular Ca^2+^ shifts and energy metabolism. This impairs mitochondrial energy metabolism, increases oxidative stress, promotes cardiomyocyte death and results in cardiac dysfunction (3, 41–44). These disruptions in Ca^2+^ homeostasis develop in response to prolonged hyperglycemia, and studies showed that impaired mitochondrial Ca^2+^ handling and its pathophysiological consequences can be reversed by insulin therapy (45, 46).

Mitochondrial morphology and DM-induced cardiomyocyte dysfunction have a reciprocal influence on each other, with DCM altering mitochondrial morphology, and altered morphology in turn promoting the progression of DCM (11). Mitochondrial morphology is controlled by a complex balance of fusion- and fission-promoting proteins. This balance is disrupted in DM, with downregulation of mitofusins 1 and 2 (MFN1 and MFN2) resulting in decreased mitochondrial fusion. MFN1 expression was found to be inversely proportional to HbA1c levels and enhancement of MFN2 in a diabetic mouse model prevented DCM progression (15, 47). ROS are able to upregulate fission proteins such as DRP1, further contributing to mitochondrial fragmentation (48).

Besides morphological changes, intracellular processes responsible for mitochondrial regeneration and quality-control were found to be impaired in DM. Mitophagy plays an important role in the healthy heart by removing dysfunctional mitochondria. Impaired mitophagy and mitochondrial biogenesis lead to persistent mitochondrial dysfunction, thereby contributing to the development and progression of DCM (49–51).

In recent years, studies have tried to elucidate the posttranscriptional changes underlying DM and its complications. Due to the association of mitochondrial function and regulation of gene expression, non-coding RNAs (ncRNAs) and RNA-binding proteins (RBPs) have emerged as key regulators of mitochondrial health and have also been implicated in the pathophysiology of DCM. With about 74% of transcribed DNA being non-coding, ncRNAs play a central role in modulating cellular responses by acting as posttranscriptional inhibitors of their target genes. While some have been shown to be critical to cellular health, others seem to contribute to various diseases, including DCM (52). Regulatory ncRNAs have a vital role in cellular homeostatic and adaptive mechanisms and are categorized according to their length: short ncRNAs are less than 200 nucleotides long and include microRNAs (miRNAs), RNAs comprising more than 200 nucleotides are referred to as long ncRNAs (lncRNAs). They are important regulators of DNA replication and transcription, as well as mRNA translation. Similarly, RBPs use RNA-binding domains to regulate gene expression through various posttranscriptional mechanisms, including alternative splicing, as well as RNA degradation, and have been shown to play a role in cardiovascular health and disease (53, 54). RBPs modulate RNA fate in a cell dependent manner to result in a tissue-specific protein repertoire and cellular function. As such, proper functioning of these intricate posttranscriptional manipulations is essential to cell health, with defects in both RBPs or RBP-regulated RNA networks showing to be central in the onset and progression of pathological disorders. Due to the involvement of RBPs in deciphering between health and disease states, RBPs have emerged as key research targets to enhance our understanding pathophysiological processes.

While their influence on mitochondrial health or dysfunction in DCM needs to be further uncovered, ncRNAs and RBPs could prove critical to understanding the exact molecular mechanisms driving DM. Here we will present the current knowledge of the roles of ncRNAs and RBPs in mitochondrial dysfunction and DCM. We explore how ncRNA/RBP-driven disruption of mitochondrial dysfunction could contribute to the development and progression of DCM, proposing key regulatory pathways and highlighting gaps in our current understanding of these mechanisms. Finally, we present established and emerging models of mitochondrial dysfunction and DM-associated vascular complications, which could provide further insights into the functions of ncRNAs and RBPs, thereby paving the way for new therapies and improved outcomes for patients suffering from DCM.

## 2. The role of ncRNAs and RBPs in mitochondrial dysfunction

### 2.1. ncRNAs in mitochondrial dysfunction

ncRNAs play a vital role in mitochondrial regulation. As 99% of mitochondrial proteins are nuclear-encoded, mitochondrial ncRNAs—called mitoRNAs—can derive from the nucleus or the mitochondria (55, 56). Adequate mitochondrial function and metabolic adaptation requires constant mito-nuclear crosstalk, which is facilitated by ncRNAs (57).

Looking at miRNAs, there are three distinct types which can influence mitochondrial function: cytoplasmic miRNAs, nuclear-encoded mitochondrial miRNAs (mitomiRs) and mitochondria-encoded mitomiRs. Cytoplasmic miRNAs have been found to play a crucial role in regulating mito-nuclear crosstalk by binding to mitochondria-associated transcripts in the cytoplasm and have been reviewed elsewhere (58). Briefly, they influence key aspects of mitochondrial function, including oxidative phosphorylation and fatty acid metabolism, as well as mitochondrial dynamics and autophagy. Cytoplasmic miR-378, for example, plays an important role in mitochondrial fatty acid metabolism, by regulating the effects of PGC-1β — a transcriptional coactivator and key regulator of mitochondrial metabolism and biogenesis. In a mouse model, miR-378 knockout increased oxidative capacity and resulted in resistance to high-fat diet-induced obesity (59). Similarly, miR-23a was shown to downregulate mitofusins and promote mitochondrial dysfunction by targeting the related transcriptional coactivator PGC-1α (60). Cytoplasmic members of the miR-30 family, on the other hand, can prevent mitochondrial fission and apoptosis by inhibiting p53 signaling and its downstream pro-apoptotic targets (61). Other miRNAs can regulate mitochondrial Ca^2+^ handling, such as miR-25, which is known to silence the expression of MCU (62). Besides acting on mitochondria-related transcripts in the cytoplasm, nuclear miRNAs and Argonaute proteins, which play a key role in miRNA-mediated gene silencing, can easily translocate into mitochondria and influence their function through downregulation of target genes (63). One example is cardiac miR-181c, which is transcribed in the nucleus and later translocates together with its silencing complex into mitochondria, where their inhibition of mitochondrial COX1 results in mitochondrial dysfunction (64). Other mitomiRs have been associated with similar regulatory mechanisms influencing mitochondrial transcripts, including miR-1 and miR-2392 (65, 66). MiR-378, which regulates mito-nuclear crosstalk and energy metabolism in the cytoplasm, is also found within mitochondria, where it was shown to inhibit the translation of ATP synthase (67, 68). While the roles of mitomiRs in mitochondrial dysfunction remain to be further uncovered, nuclear- and mitochondrial-encoded miRNAs, both within the cytoplasm and mitochondria, undoubtedly play a central role regulating mitochondrial metabolism and dynamics and might be important drivers of regulatory dysregulations see in mitochondrial dysfunction.

LncRNAs can promote or prevent mitochondrial dysfunction through multiple mechanisms and have been implicated in various cardiovascular diseases. Interestingly, Yang et al. found expression profiles of lncRNAs, but not mRNAs or miRNAs, to characterize various pathologies of the failing heart. Much like miRNAs, lncRNAs can be encoded by nuclear or mitochondrial DNA and act on mitochondria-related transcripts both in the cytoplasm and within mitochondria (58). Some lncRNAs are known to dysregulate mitochondrial energy metabolism, such as the lncRNA AK055347, which controls the expression of ATP synthase in the heart (69). Others regulate the complex balances between mitochondrial fission and fusion as well as mitophagy and biogenesis. LncRNA HOTAIR regulates mitoribosomal proteins and its inhibition has been shown to result in mitochondrial dysfunction (70, 71). While lncRNA H19 reduces the expression of fusion protein MFN2 through miR675-mediated gene silencing, lncRNA CARL sponges miR-539, thereby suppressing mitochondrial fission and apoptosis (72, 73). Other lncRNAs have been shown to regulate gene expression by inhibiting miRNA-associated gene silencing, including CEROX1, which increases expression and activity of complexes I and IV by preventing their miR-488-3p-driven downregulation (74). Similarly, lncRNA KCNQ1OT1sponges miR-378 and prevents its inhibition of ATP synthase translation (67). Other lncRNAs interact with gene promoter regions, such as TUG1, which has been shown to induce PGC-1α and regulate mitochondrial bioenergetics (75). LncRNA UIHTC has also been shown to improve mitochondrial function in cardiomyocyte by enhancing PGC-1α expression (76). Though the exact effects and underlying mechanisms of many lncRNAs remain poorly understood, they have been found to play important regulatory roles in mitochondrial health and dysfunction, potentially contributing to the pathogenesis of cardiovascular and other diseases.

**Figure 2:**
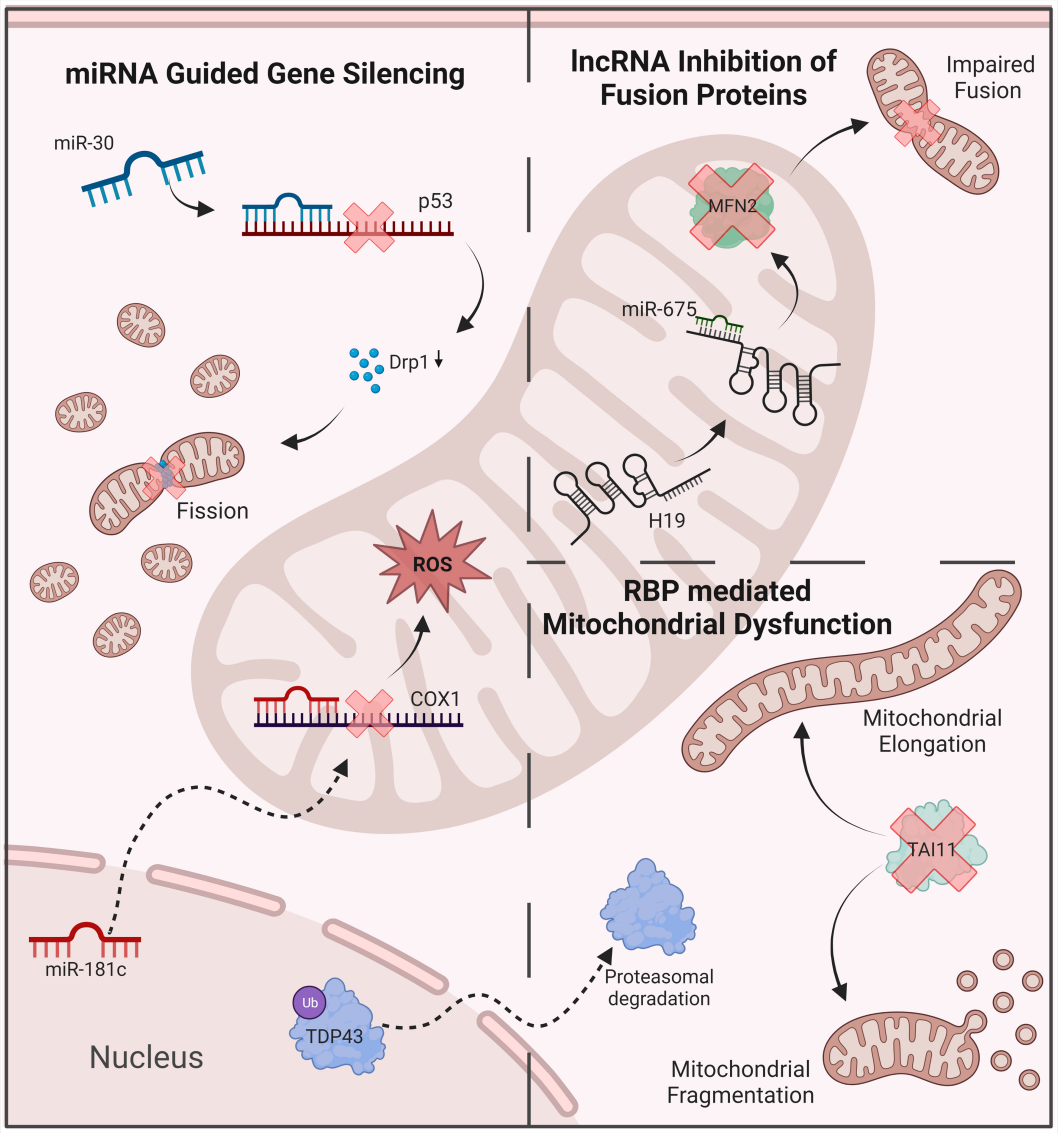
Examples of ncRNAs anr RBPs’ involvement in mitochondrial dysfunction. MiRNA-mediated gene silencing contributes to the disruption of oxidative balance and mitochondrial dynamics. LncRNas inhibit key regulatory proteins, including mitofusin 2. RBPs disrupt mitochondrial dynamics, leading to abnormal morphology and function. ROS, reactive oxygen species; MFN2, mitofusin 2; DRP1, dynamin-related protein 1.

Other ncRNAs have been shown to play crucial roles in mitochondrial function. Ro et al., for example, identified mitochondrial small RNAs (mitosRNAs) which are derived from sense transcripts of mitochondrial genes and seem to regulate mitochondrial gene expression (77). Studies also identified hundreds of mitochondria encoded circRNAs (mecciRNAs) (78). Similar to lncRNAs, circRNAs are able to sponge miRNAs, thereby increasing the translation of their target genes (79). CircRNA 0000495, for example, is able to sponge and inhibit the silencing action of miR-488-3p, which is known to target electron transport chain complexes (74, 80). While circRNAs seem to play an important physiological function and are involved in various mitochondrial functions, their impact on mitochondrial dysfunction in diseases such as DCM remains unclear (81). Besides ncRNAs, other posttranscriptional regulators of gene expression are known to be involved in mitochondrial dysfunction, including RBPs (Figure 2).

### 2.2. RBPs in mitochondrial dysfunction

Despite the involvement of RBPs in various disease states, the effect of mitochondrial dysfunction on RBP involvement in cardiac phenotypes has not been fully investigated. Nevertheless, a connection between RBPs and oxidative stress has been uncovered in the recent years, albeit with a focus on neurological diseases, primarily Amyotrophic lateral sclerosis (ALS). Although studied under different physiological conditions, RBPs identified to have oxidative stress induced posttranscriptional modifications similarly have known fundamental roles in cardiac cells also. TAR DNA binding protein 43 (TDP-43), for example, is a versatile RNA/DNA binding protein involved in RNA-related metabolism. Moreover, TDP-43 is known to interact with several mitochondrial proteins and is thought to have a role in the stabilization of mitochondrial transcripts (82, 83). Dysfunctional TDP-43 has therefore been linked to various mechanisms of mitochondrial dysfunction, previously reviewed elsewhere (84). In brief, overexpression of TDP-43 results in abnormal mitochondrial morphology, disrupted mitochondrial dynamics and altered mitochondrial-ER contacts (85–87). Furthermore, a recent study identified the mitochondrial DNA release as a result of TDP-43 expression to drive an inflammatory response mediated via the cGAS-STING pathway (88). Knockdown, on the other hand, reduced the number of mitochondria, mitochondrial membrane potential as well as the expression of mitochondrial transcripts (89). Inhibition of TDP-43 mediated dysfunction may serve as a beneficial therapeutic approach in certain disease states (90).

Oxidative stress has been shown to cause the acetylation and cysteine oxidation of TDP-43, both of which result in increased TDP-43 aggregation as well as its phosphorylation (91–95). The phosphorylation of TDP-43 is heavily associated with deleterious functional changes including mis-localization, decreased turnover, changes in solubility, and altered splicing activity, to name a few (96). Mutations in TDP-43, including its aggregation and depletion, is heavily associated with ALS. Studies to investigate the effect of loss of function of TDP-43, a consequence of phosphorylation, have also uncovered cardiovascular complications, of which further investigation is needed. For example, a recent study investigating the loss of TDP-43 function in mice found these mice to have short lifespans, accredited to cardiac failure due to TDP-43 knockdown mice having enlarged hearts (97). Similarly, in zebrafish, silencing of TDP-43 resulted in early death attributed to severely reduced blood circulation and resulted in the mis-patterning of blood vessels, alongside muscle degeneration (98). Since these studies, phosphorylated TDP-43 has shown to aggregate within cardiac muscle as well as blood vessels (99, 100). Likewise, the RBP TAI1, has been found to undergo cysteine oxidation in response to oxidative stress (101). TAI1 is commonly known for regulating alternative splicing of select pre-mRNAs and promoting the assembly of stress granules, however, roles for TAI1 in mitochondria fission have also been identified. Moreover, downregulation of TAI1 results in mitochondrial elongation or overexpression and increased abundance of proteins involved in mitochondrial fission (102, 103). Similarly, the RBP HuD, known to regulate MFN2, is downregulated in diabetes contributing to mitochondrial dysfunction (104). It is widely accepted that interruption of mitochondrial fission and fusion contributes to cardiomyopathies, further investigation of the impact mitochondrial dysfunction has on RBPs regulating fission/fusion could therefore provide further insight. Likewise, as the prevalence of mitochondrial dysfunction in cardiomyopathies is already established, as in the case of other diseases, the evaluation of oxidative stress on RBPs in cardiomyopathies will undoubtedly identify novel pathogenic mechanisms and potential therapeutic targets.

## 3. The role of ncRNAs and RBPs in diabetic cardiomyopathy

### 3.1. ncRNAs in diabetic cardiomyopathy

Multiple miRNAs have been found to be differentially expressed in *in vitro* and animal models of DCM (Table 1). Diao et al. investigated miRNA expression in streptozotocin-induced diabetic mice and identified ten up- and six downregulated miRNAs which were linked to genes associated with cardiac hypertrophy and fibrosis, including TGFB3 and COL1A1 (132). Similarly, 43 miRNAs were found to be differentially expressed in a rat model of DCM. This model showed characteristic features of DCM, including changes in sarcomeric and mitochondrial structure, as well as increased oxidative stress and contractile dysfunction. Antioxidant treatment reversed these pathological features and restored the levels of previously downregulated miRNAs, including miR-1 and miR-133a (105). MiR-1 and miR-133a are highly expressed in healthy cardiomyocytes and have known cardioprotective properties. Their downregulation in diabetic myocardium results in increased cardiac autophagy and hypertrophy, which are commonly seen in heart failure (111). Overexpression of miR-133a, on the other hand, protects from cardiac fibrosis by downregulating TGF-β1 and preventing the phosphorylation of ERK1/2 and SMAD2 (112). In the same DCM rat model, miR-21, which is known to promote high-glucose induced cardiac fibrosis, was found to be upregulated (105, 120). Similarly, miR-155 is known to enhance inflammatory signaling and contribute to adverse inflammatory responses in cardiovascular diseases (133). Therapeutic downregulation of miR-155 in ovariectomized diabetic mice led to a reduction in cell apoptosis and restoration of cardiac function (134). Other miRNAs are known to prevent pyroptosis but are downregulated in DCM. miR-9 levels, for example, are reduced in hyperglycemic cardiomyocytes. miR-9 targets ELAV-like protein 1 (ELAVL1) — a mRNA-stabilizing protein which enhances TNF-ɑ-induced cardiac cell death. In an *in vitro* model, upregulation of miR-9 prevented cardiomyocyte pyroptosis in response to hyperglycemia (106).

**Table 1:** Dysregulated miRNAs identified in models of diabetic cardiomyopathy.

Name	Expression	Proposed role/mechanism	References
miR-1	Down	Regulation of cardiac contractility through targeting sarcoplasmic reticulum protein junctin	(105)
miR-9	Down	attenuates ELAVL1 activity and inhibits cardiomyocyte pyroptosis	(106)
miR-15a/b	Down	Suppresses fibrosis through TGFβR1 and CTGF inhibition	(107)
miR-22	Down	Promotes Sirt 1 expression and attenuates oxidative stress	(108)
miR-30c	Down	Attenuates hypertrophy and reduces autophagy through inhibition of various factors including Cdc42/Pak1 and BECN1	(109, 110)
miR-133a	Down	Suppresses cardiac hypertrophy and fibrosis via decreased TGF-β1, ERK1/2 and SMAD2 signaling	(111, 112)
miR-144	Down	Inhibits Nrf-2 and promotes oxidative stress	(113)
miR-146a	Down	Inhibits inflammatory mediators with cardioprotective effect	(114)
miR-150	Down	Prevents p300-mediated cardiomyocyte hypertrophy	(115)
miR-200b	Down	Cardioprotective through inhibition of p300 and other mediators	(116)
miR-373	Down	Suppresses hypertrophy through MEF2C inhibition	(117)
miR-1	Up	Inhibits IGF-1 resulting in mitochondrial dysfunction and apoptosis	(118, 119)
miR-21	Up	Promotes cardiac fibrosis through DUSP8 suppression and JNK/SAPK and p38 signaling pathway activation	(120)
miR-29	Up	Inhibits MCL-1 resulting in cardiac structural damage	(121)
miR-30d	Up	Inhibits Foxo3a expression, increases caspase-1 and promotes pyroptosis	(122)
miR-34a	Up	Inhibits Bcl-2 resulting in increased apoptosis	(123)
miR-141	Up	Inhibits Slc25a3 and promotes mitochondrial dysfunction	(124)
miR-193-5p	Up	Inhibits IGF-2 and promotes angiogenesis	(125)
miR-195	Up	Inhibits BCL-2 and Sirt1 expression, promoting hypertrophy and apoptosis	(126)
miR-206	Up	Promotes cardiomyocyte apoptosis through Hsp60	(119)
miR-301a	Up	Causes electrical remodeling though inhibition of voltage-gated potassium channel Kv4.2	(127)
miR-320	Up	Inhibits IGF-1 and impairs angiogenesis	(128)
miR-451	Up	Promotes hypertrophy through inhibition of CAB39 and suppression of the LKB1/AMPK pathway	(129)
miR-483-3p	Up	Inhibits IGF-1 and promotes cardiomyocyte apoptosis	(130)
miR-503	Up	Inhibits Nrf-2-mediated expression of antioxidant enzymes	(131)

Other miRNAs modulate cellular metabolism and promote or prevent the shifts in molecular mechanisms underlying DCM pathogenesis. As described, PPARα signaling facilitates the increase in fatty acid metabolism and contributes to lipotoxicity in diabetic cardiomyocytes. PGC-1β induces PPARα and is targeted by miR-30c. Overexpression of miR-30c resulted in reduced transcriptional activity of PPARα and reversed the metabolic shift towards increased uptake and use of fatty acids (135). Conversely, miR-503 might contribute to DCM development through inhibiting Nrf-2 mediated expression of antioxidant enzymes. In a diabetic rat model, cardiomyocytes exhibited low levels of Nrf2 and increased expression of miR-503 (131). MiR-451 is also upregulated in response to high fatty acid levels in the hearts of Type 2 diabetic mice. Knockout of miR-451 in this model reduced lipotoxicity and improved features of DCM, including cardiac hypertrophy (129). Similarly, miR-195 is increased in mouse models of Type 1 and Type 2 DM and its knockdown was shown to improve cardiac function while reducing oxidative stress and hypertrophy (126). Decreased expression of miR-30c in DCM, on the other hand, results in increased hypertrophy (109). Other miRNAs, such as miR-143, are regulating insulin action and may contribute to insulin resistance seen in Type 2 DM (136).

**Figure 3:**
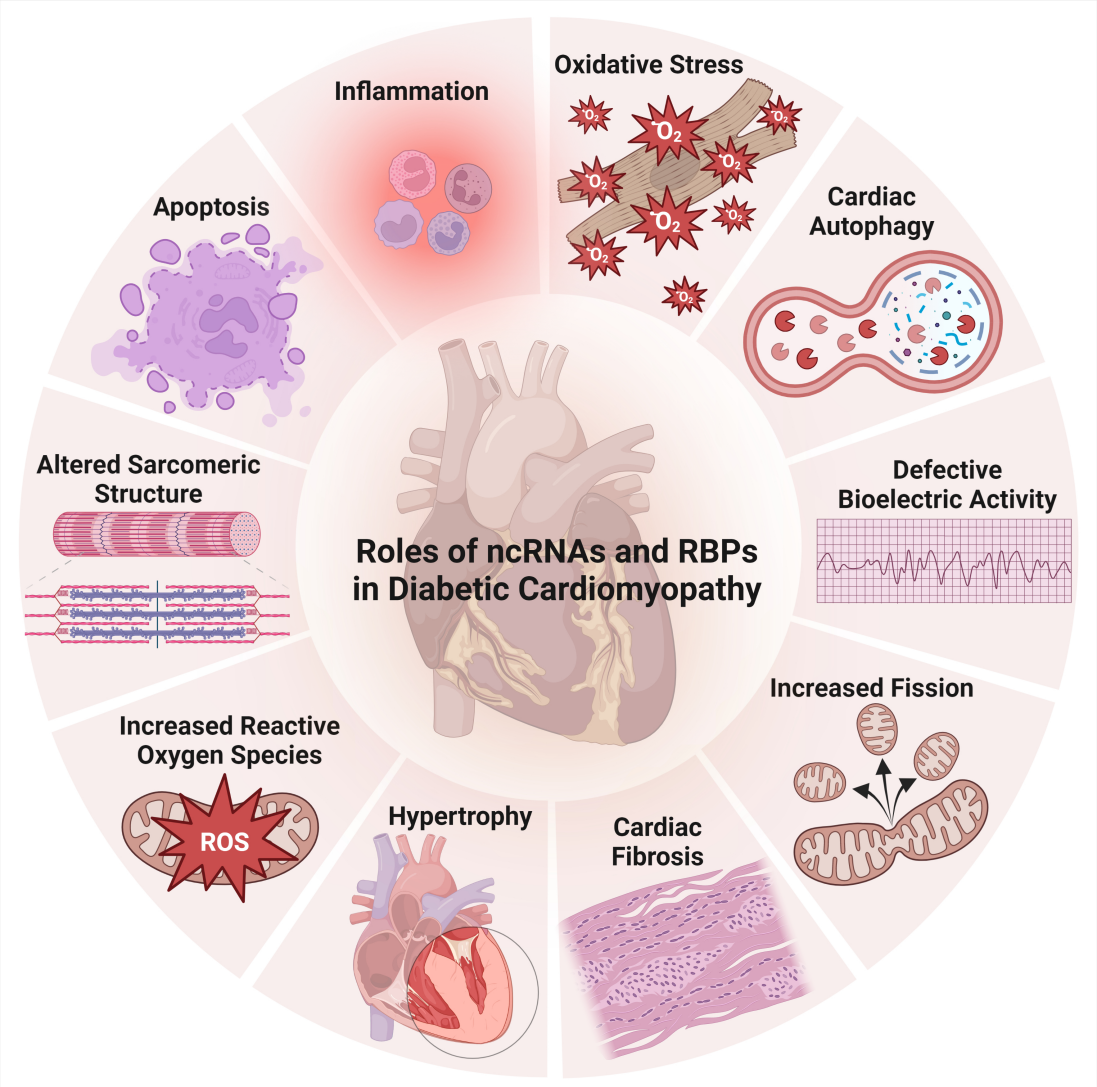
Aspects of ncRNAs and RBPs’ involvement in diabetic cardiomyopathy.

Similar to miRNAs, lncRNAs have been shown to posttranscriptionally regulate key cellular mechanisms, including energy metabolism as well as inflammation, and have been found to be implicated in the pathophysiological changes seen in DCM (Table 2, Figure 3). In a diabetic mouse model, DCM development was accompanied by significant changes in lncRNA expression. Differentially expressed lncRNAs were found to be associated with myofilament development and motion, as well as inflammation, immunity and apoptosis (152). The lncRNA H19 is a precursor of miR-675, which targets VDAC1—a voltage-gated ion channel and critical component of mitochondria-mediated apoptosis. H19 regulates cardiomyocyte apoptosis through VDAC1 inhibition. In DCM, however, it is found to be downregulated, leading to increased apoptosis, which eventually results in cardiac dysfunction. H19 overexpression was found to reduce oxidative stress, inflammation and apoptosis, as well as improve left ventricular function (138, 139). Similarly, DCM-associated upregulation of lncRNA MALAT1 results in increased cardiomyocyte apoptosis. Downregulation of MALAT1 in a DCM rat model improved cardiac function by attenuating apoptosis (146). Other lncRNA are modulating cardiac fibroblast-to-myofibroblast differentiation and are involved in the fibrotic response seen in DCM. The expression of lncRNA CRNDE negatively correlates with profibrotic genes, for example, and its overexpression has been shown to reduce cardiac fibrosis and improve contractile function in DCM mice (143).

**Table 2:** Dysregulated lncRNAs identified in models of diabetic cardiomyopathy.

Name	Expression	Proposed role/mechanism	References
GAS5	Down	Prevents inflammasome-mediated pyroptosis through sponging of miR-34b-3p	(137)
H19	Down	Inhibits autophagy and apoptosis through DIRAS3/mTOR and miR-675/VDAC1 signaling regulation	(138, 139)
HOTAIR	Down	Increases SIRT1 expression though sponging of miR-34a resulting in decreased oxidative stress and inflammation	(140)
AK081284	Up	Increases collagen and TGF-β1 expression	(141)
ANRIL	Up	Regulates the expression of extracellular matrix proteins	(142)
CRNDE	Up	Attenuates cardiac fibrosis through inhibition of Smad3-mediated cardiac myofibroblast differentiation	(143)
DCRF	Up	Induces autophagy by preventing miR-551b-5p-mediated PCDH17 suppression	(144)
KCNQ1OT1	Up	Induces pyroptosis through miR-214-3p/caspase-1 signaling	(145)
MALAT1	Up	Induces inflammation through increased inflammatory cytokine expression	(146)
MEG3	Up	Suppresses miR-145 and promotes cardiomyocyte apoptosis	(147)
MIAT	Up	Induces apoptosis through miR-22-3p sponging and increased DAPK2 expression	(148)
NEAT1	Up	Promotes apoptosis through miR-140e5p sponging	(149)
NONRATT007560.2	Up	Increases ROS production and apoptosis	(150)
TUG1	Up	Promotes hypertrophy and diastolic dysfunction through inhibition of miR-499-5p	(151)

Adding another layer of posttranscriptional regulation of gene expression, some lncRNAs are inhibiting gene silencing by sponging miRNAs. HOTAIR lncRNAs acts by sponging miR-34a, thereby inhibiting inflammation and apoptosis. Its expression is downregulated in DCM and overexpression in a diabetic animal model has been shown to reduce cardiac inflammation, oxidative injury and cardiomyocyte apoptosis (140). Other lncRNAs are upregulated in DCM, leading to increased miRNA-sponging and reduced silencing of target genes. MIAT lncRNA was found to sponge miR-22-3p in a DM rat model, and its upregulation in DCM promoted excessive cardiomyocyte apoptosis (148). Similarly, overexpression of lncRNA KCNQ1OT1 in DCM inhibits the miR-214-3p-regulated silencing of caspase-1, resulting in increased inflammatory signaling and pyroptosis (145).

Other ncRNAs, such as circRNAs are known to be involved in the posttranscriptional signaling shifts seen in DM and its complications, including DCM (Table 3) (153, 154). Several circRNAs which are upregulated in DCM are known to promote myocardial fibrosis by regulating miRNAs and their downstream targets. Sponging of miRNAs by these circRNAs results in upregulation of associated miRNA target genes, including TGF-β1 and collagen (157, 160, 161). Similarly, circRNA CACR, which is upregulated in high-glucose-treated cardiomyocytes and in the serum of DM patients, promotes pyroptosis by sponging miR-214-3p, leading to an increase in caspase-1 activity (156). Conversely, circRNA DICAR can alleviate pyroptosis and other hallmarks of DCM. However, DICAR was found to be reduced in both diabetic mouse hearts and the blood of DM patients (155).

**Table 3:** Dysregulated circRNAs identified in models of diabetic cardiomyopathy.

Name	Expression	Proposed role/mechanism	References
circDICAR	Down	Prevents cardiomyocyte pyroptosis, hypertrophy and fibrosis	(155)
circCACR	Up	Promotes pyroptosis through miR-214-3p sponging and caspase-1 activation	(156)
circHIPK3	Up	Suppresses miR-29b-3p resulting in increased collagen expression and fibrosis	(157)
circ_000203	Up	Suppression of miR-26b-5p increases CTGF and collagen expression	(158)
circ_0071269	Up	Sponges miR-145 resulting in inflammation and pyroptosis	(159)
circ_010567	Up	Suppresses miR-141-mediated TGF-β1 inhibition, leading to fibrosis	(160)

Altogether, these results show that while many ncRNAs have been implicated in DCM, their complex mechanistic interactions and modes of regulation remain to be further studied and better understood.

### 3.2. RBPs in diabetic cardiomyopathy

Numerous proteomic studies have demonstrated the depth of RBP involvement in cardiac health and disease through isolating hundreds of cardiomyocyte-specific RBPs (162, 163). Moreover, assessment of RBP splicing patterns within adult hearts has been heavily linked to cardiomyocyte function. Specifically, the expression of crucial splicing factors SF1, ZRSR2, SRSF4, and SRSF5 is downregulated in dysfunctional cardiomyocytes. Within the heart, binding of RBP CIRP to mature RNAs has been shown to enhance the translation of KCND2 and KCND3, inhibition of which results in reduced voltage-gated potassium channel function and defective bioelectric activity (164). Likewise, RBP MBLN1 was shown to regulate the expression of voltage-gated sodium channel SCN5A (165). Moreover, RBP PCBP2 was found to promote GPR56 mRNA degradation in cardiomyocytes, inhibiting angiotensin II-induced hypertrophy (166).

Although the function for the majority of cardiac specific RBPs remains unknown, several studies have revealed dysfunctional RBPs to contribute to cardiomyopathies, in particular diabetic cardiomyopathy (Table 4). Whilst this review focuses on critical RBPs which in the recent years have been heavily implicated in diabetic cardiomyopathy, it is noteworthy to mention a role for RBPs within the blood vascular system and cardiovascular disease onset and progression has also been established and previously reviewed elsewhere (54). Human genetic studies have revealed polymorphisms and mutations in RBPs to be linked with diabetes, as such the role of RBPs in diabetic cardiomyopathy has been a recent topic of interest. Assessment of diabetic hearts revealed the RBP CUG-BP, also referred to as CELF1, a highly conserved RBP that regulates alternative splicing, polyadenylation, mRNA stability, and translation, for instance, to be upregulated (169). Moreover, in mice, overexpression of CUG-BP caused dilated cardiomyopathy and heart failure and interestingly a reactivation of embryonic splicing patterns (169–172). Accordingly, numerous studies have also isolated fetal specific RBPs in adult heart failure tissues, indicating that during heart failure fetal specific RBPs are reactivated and suggesting a role for fetal RBPs in cardiac disease development, including in DM (173). Thus, suggesting a role for fetal RBPs in cardiac disease development, including in diabetes.

**Table 4:** Dysregulated RBPs identified in models of diabetic cardiomyopathy.

Name	Expression	Proposed role/mechanism	References
LIN28a	Down	Protects against DCM by inhibiting Mst1 and PKA/ROCK2 signaling	(167, 168)
CUG-BP	Up	Alternative splicing of target genes might contribute to DCM pathogenesis	(169)
ELAVL1	Up	Induces pyroptosis via NLRP3, caspase-1 and IL-1β signaling	(106)
RBFOX2	Up	Alternative splicing of target genes might contribute to DCM pathogenesis	(169, 170)

Moreover, within diabetic endothelial cells, overexpression of CUG-BP enhances the expression of the alternatively spliced isoform QKI-7, orchestrating endothelial cell dysfunction (174). QKI-7 belongs to the Signal Transduction and Activation of RNA (STAR) RBP Quaking family, alternative splicing of which generates three major isoforms known as QKI-5, QKI-6 and QKI-7. Both major isoforms QKI-5 and QKI-6 were shown to have pivotal roles in regulating and maintaining cardiovascular health, deficiency of which triggers embryonic lethality (175). In addition, studies have demonstrated both QKI-5 and QKI-6 to be anti-apoptotic in cardiomyocytes, favoring cell survival via the elevation of pro-apoptotic factors during cardiac injury (176). Furthermore, QKI-5 was recently shown to be integral to the regulation of cardiac myofibrillogenesis and its deficiency promotes apoptosis and atrophy in cardiomyocytes (177, 178).

Another RBP known to regulate many genes involved in cardiac function via alternative splicing is RBFOX2. In multiple animal models, a downregulation of RBFOX2 has been shown to lower heart rate, cause myofibrillar disarray and result in heart failure (179, 180). Moreover, in individuals with cardiac disease, mutations in RBFOX2 have been identified (181). Unsurprisingly, a role for RBFOX2 has also been identified in diabetic cardiomyopathies. Although studies have shown RBFOX2 levels to be high in diabetic hearts, its alternative splicing activity is low (169, 170). Like the alternative splicing of the QKI family, analysis of RBFOX2 in the diabetic heart revealed diabetic hearts to express a dominant negative alternatively spliced isoform of RBFOX2 responsible for blocking RBFOX2-mediated alternative splicing (170). Further analysis of the mis-spliced transcripts in diabetic hearts revealed 73% to have RBFOX2-binding sites, including genes associated with the expression of cytoskeleton and intracellular calcium handling (170). Accordingly, RBFOX2 dysregulation contributes to diabetic complications in the heart. A reduction of LIN28, an RBP predominantly known for its roles in promoting pluripotency, in cardiomyocytes has shown to decrease contractile function and cell death (167). LIN28 was found to be significantly reduced in diabetic hearts and exacerbate cardiac symptoms observed through a decrease in left ventricular ejection fraction, increased apoptotic index and mitochondrial dysfunction (167, 182). Overexpression of LIN28, however, prevented cardiomyopathy in diabetic mice, demonstrating its protective role in cardiac function (167, 168). Comparably, knockdown of the RBP ELAVL1, a known major contributor to diabetic complications, which has also been found to be upregulated in diabetic hearts and correspond with cardiomyocyte death, resulted in smaller infarct size and fibrosis area following a myocardial infarction (106, 183). Extensive evidence therefore demonstrates a primary role for RBPs and their dysregulation in the development and progression of diabetic cardiomyopathies; revelations of such roles may aid the development of novel therapeutic interventions.

## 4. Discussion

Mitochondrial dysfunction has emerged as a key driver of cardiovascular diseases, including DCM. While mitochondria are mainly known for their metabolic roles and properties, they are integral to other important cellular processes, including oxidative balance, Ca^2+^ handling and apoptosis. Hence, mitochondrial dysfunction goes far beyond alterations of energy metabolism and manifests in disruptions of both intra- and extramitochondrial cellular mechanisms. Mitochondrial dysfunction has been thoroughly studied and implicated in DCM pathogenesis, its hallmarks including a shift towards increased fatty acid uptake and oxidation leading to decreased cardiac efficiency, increased oxidative stress causing mitochondrial damage, disruption of Ca^2+^ homeostasis resulting in contractile dysfunction as well as imbalance in fission and fusion giving rise to mitochondrial fragmentation (3, 11, 12).

In recent years, ncRNAs and RBPs were studied in many diseases and have been implicated in both mitochondrial dysfunction and DCM. Some seem to serve physiological or protective functions and their downregulation is found to be associated with various disease states. Conversely, others contribute to the disruption of mitochondrial function and pathophysiological changes seen in DCM (58, 81, 82, 132, 152, 163). This review aimed to collect and newly synthesize current insights into ncRNA and RBP involvement in mitochondrial dysfunction in the context of DCM.

Many ncRNAs and RBPs which are known to play a key role in mitochondrial health and dysfunction, have also been implicated in DCM (Table 5). For the majority of these, their impact on mitochondrial health or dysfunction has been assessed in diseases other than DCM—most studies using cancer-related *in vitro* models—and their specific function in DCM remains to be uncovered. Only for few ncRNAs, including miR-1 and miR-141, a specific role and associated mechanistic pathway impacting on mitochondrial dysregulation in the context of DCM has been determined (118, 124).

**Table 5:** ncRNAs and RBPs involved in mitochondrial health/dysfunction and found to be differentially expressed in DCM.

Type	Name	Target	Mechanism in mitochondrial dysfunction	Expression in DCM	References
miRNA	miR-1	IGF-1	Prevents IGF-1-mediated restoration of mitochondrial membrane potential resulting in release of cytochrome-c	Down/Up	(118)
	miR-9	BCL2L11	Prevents apoptosis through BCL2L11 inhibition	Down	(184)
	miR-22	SIRT1	Promotes mitochondrial damage through SIRT1/PGC-1α inhibition	Down	(185)
	miR-34a	SIRT1	Promotes apoptosis through SIRT1 inhibition	Up	(186)
	miR-141	SLC25A3	Suppresses SLC25A3, modulating ATP production and ATP synthase activity	Up	(124)
	miR-195	MFN2	Disrupts mitochondrial morphology and function through targeting MFN2	Up	(187)
lncRNA	ANRIL	BCL2	Promotes cytochrome-c release and apoptosis through BCL2 inhibition	Up	(188)
	GAS5	BCL2	Promotes apoptosis through BCL2L4/BCL2L7 signaling	Down	(189)
	H19	miR-675	Prevents BCL2/VDAC1-mediated apoptosis through miR-675 induction	Down	(138)
	HOTAIR	UQCRQ, MICU1	Maintains mitochondrial function through UQCRQ regulation and prevents MICU1-mediated apoptosis	Down	(71, 190)
	MEG3	BCL2	Promotes cytochrome-c release and apoptosis through BCL2 inhibition	Up	(191)
	MIAT	TSPO	Promotes TSPO-mediated apoptosis	Up	(192)
	TUG1	PGC-1α	Improves mitochondrial bioenergetics through PGC-1α enhancement	Up	(75)
RBP	CUG-BP	BCL2L4/8, JUND	Prevents apoptosis through mediating the decay of BCL2L4, BCL2L8 and JunD	Up	(193)
	ELAVL1	DRP1	Promotes mitochondrial fragmentation through regulation of fission protein DRP1	Up	(194)
	LIN28a	LARS2	Promotes mitochondrial dysfunction by inhibiting LARS2/PGC-1α/Nrf2 signaling	Down	(195)
	RBFOX2	SLC25A4	Contributes to mitochondrial health through mitochondrial gene expression regulation	Up	(196)

Overall, there have been few studies looking at unifying pathways that would link ncRNA/RBP-associated dysregulation of mitochondrial function to DCM development and progression. While this fascinating link remains to be uncovered, the required research is complicated by the complex mechanisms and interactions underlying ncRNA/RBP-driven posttranscriptional regulation of gene expression. The associated processes are multifaceted and comprise different layers of regulation, impacting every stage of gene expression, including transcription, splicing and translation.

MiRNAs, such as miR-675, can regulate mitochondrial function through inhibition of multiple key regulators, including the fusion protein MFN2 and the anion channel VDAC1 (138, 197). MiR-675's potential role in DCM highlights the complex and sometimes contradicting mechanisms that need to be understood in order to fully appreciate the underlying regulatory pathways. Studies observed downregulation of miR-675 in the context of hyperglycemia which led to increased cardiomyocyte apoptosis. While excessive cardiomyocyte apoptosis can be seen in cardiomyopathies, miR-675 downregulation also results in increased expression of MFN2, which is contrary to what many studies observed in DM. This not only highlights the complexity of one protein, such as MFN2, being targeted by multiple posttranscriptional regulators, but might also exemplify the difficulty of deciphering the exact role of each player in the coexisting health-protecting and disease-promoting processes. While some expression changes might actively promote DCM development and progression, others might be a protective response aimed at maintaining cellular homeostasis and function. Additionally, effects of molecular expression changes might differ in the acute and chronic setting, as seen in other cardiovascular diseases (198). Some protective responses in the context of acute hyperglycemia might become pathological if maintained for too long. With multiple targets, both within and outside of mitochondria, miRNAs can be involved in mitochondrial and extra-mitochondrial processes, such as miR-214, which targets MFN2, but also the tumor suppressor protein PTEN and various other genes (199, 200). Furthermore, lncRNAs and other ncRNAs can influence and modulate miRNA action. MiR-214, for example is known to be inhibited by lncR KCNQ1OT1, which promotes cardiomyocyte pyroptosis and is upregulated in DM (145). However, the role of KCNQ1OT1 and many other miRNA-regulating ncRNAs in mitochondrial dysfunction has never been explicitly investigated. Considering that gene expression changes are not dependent on a single mediator, but are influenced by the complex, interconnected relationships of various posttranscriptional regulators, it is not surprising that the exact role of individual ncRNAs or RBPs in mitochondrial dysfunction and DCM is difficult to ascertain. Nevertheless, the emerging evidence linking ncRNAs and RBPs with mitochondrial dysfunction and DCM independently, justifies further studies into unifying pathways and mechanisms that could underly mitochondrial dysfunction in DCM.

Looking beyond these complex regulatory mechanisms, certain genes seem to emerge as particularly prominent targets. These include regulators of mitochondrial metabolism, ion channels, and mitochondrial fusion/fission proteins. PGC-1α/β, for example, seem to be targeted by various ncRNAs associated with both mitochondrial dysfunction and DCM (60, 135, 201). Given their central role in mediating the uptake and use of fatty acid, their crucial role in DCM-associated metabolic shifts appear plausible. Additionally, they have been shown to impact on proinflammatory and prooxidant signaling, further highlighting their potential importance for the development of mitochondrial dysfunction in the context of DCM (198, 202). Similarly, mitofusins appear to be targeted by various ncRNAs and RBPs implicated in DCM, linking DM-associated pathophysiological changes to disrupted mitochondrial dynamics.

Since ncRNAs and RBPs are secreted into the systemic circulation and their blood concentrations have been shown to differ in patients compared to heathy controls, they have been proposed as circulating biomarkers for various diseases. For instance, a large number of cardiac and non-cardiac miRNAs, including miRs 1 and 133a, have shown biomarker potential due to their differential serum concentrations in cardiovascular disease (203). Various ncRNAs have also been proposed as biomarkers for Type 2 DM and its complications, such as diabetic retinopathy (204–206). In the context of DCM, lncRNA serum levels were found to be directly associated with diastolic dysfunction and cardiac remodelling in patients with Type 2 DM (207).

Furthermore, some circulating ncRNAs have been shown to correlate with commonly used clinical markers, such as serum glucose and HbA1c, in patients with Type 1 and 2 DM (208, 209). However, there is an ongoing need for prospective studies which track the expression levels of ncRNAs or RBPs and correlate those with DCM severity and progression in order to adequately assess the potential of these mediators as diagnostic or prognostic biomarkers.

Therapies trying to build upon these insights, might focus on posttranscriptional regulators, including ncRNAs and RBPs, or their targets, such as PGC-1α/β or MFN1/2. Deciding on the most promising or effective therapeutic target is made difficult by the fact that each regulator has multiple targets, and each target has multiple regulators. Potential RNA-based therapies can either use ncRNAs as therapeutic agents to re-establish physiological expression levels of key genes, or directly target ncRNAs known to be involved in pathogenic processes. While multiple such agents have been approved or are currently undergoing clinical trials, there remain challenges in therapeutically using or targeting ncRNAs and other transcriptional regulators, which include agent immunogenicity, target specificity, and effective delivery (210). To improve our understanding and allow therapeutic application of new insights, further studies need to uncover the exact mechanisms and identify key drivers underlying mitochondrial dysfunction in the context of DCM.

## 5. Future work

While *in vivo* animal models, such as obese (ob/ob) or diabetic (db/db) mice, are a great source of insight into the pathophysiological manifestations of DCM and the effect of interventions, such as the administration of insulin, the further investigation and thorough understanding of the underlying molecular mechanisms require adequate and reliable *in vitro* models. Such models should display key features of DCM, including insulin resistance, increased fatty acid metabolism and oxidative stress, as well as disrupted Ca^2+^ handling and contractile dysfunction (211). In recent years, stem cell models have emerged as a promising tool for studying DM and its vascular complications.

Other *in vitro* models have been frequently used in the past, each, however, with its own challenges. While H9C2 rat cardiac cells or HL-1 immortalized mouse cardiomyocytes are easy to culture and study, their applicability in the context of DCM is limited due to significant differences in their expression of key cardiac genes and phenotypic features. Primary adult cardiomyocytes, on the other hand, are difficult to culture and while neonatal cells are easier to maintain, their phenotype remains immature (212, 213).

Induced pluripotent stem cells (iPSCs)-derived cardiomyocytes have been used to study various cardiovascular diseases, including myocardial hypertrophy, arrythmias and cardiomyopathies, with some of their features, such as being of human origin as well as the possibility of generating patient-specific models, making them a particularly useful model system (214, 215). The use of models comprising a single cell type, such as cardiomyocytes, offers a chance to study cell specific mechanisms, but cannot capture the complex interactions of different cells types seen *in vivo*. Therefore, organoids and other three-dimensional models using multiple cell types, are an interesting and rapidly developing resource for future studies into the mechanisms underlying vascular complications in DM (216). While iPSC-derived cardiomyocytes have been used as models of DCM and offer fascinating insights into disease- and patient-specific molecular changes, further validation and characterization of differentiation protocols are needed to ensure adequate reproducibility and applicability (214, 217).

iPSCs have also been widely used to model mitochondrial diseases, since their metabolic profile allows for manipulation of mitochondrial function while preserving cell viability (218). Assessing key mitochondrial properties, such as oxidative capacity, Ca^2+^ handling and fission/fusion, in these cells provide further insights into the mitochondrial changes resulting from and possibly contributing to disease development and progression. Li et al., for instance, investigated mitochondrial dysfunction in hypertrophic cardiomyopathy using iPSC-derived cardiomyocytes. Measuring mitochondrial morphology, mitochondrial membrane potentials and Ca^2+^ flux, they were able to correlate mitochondrial functional disruptions with pathophysiological changes, including electrophysiological abnormalities (219).

Similar iPSC-models could provide valuable insights when investigating the role of ncRNAs and RBPs in regulating mitochondrial health and dysfunction in DCM. Their unique features, including the patient-specific genetic profile, make iPSC-based models suitable to study aspects of DCM which have been challenging to determine using other *in vitro* models, such as the importance of ncRNA/RBP expression differences in type 1 and type 2 DM-associated DCM and their potential contribution to variabilities in underlying pathological mechanisms. Building on the current understanding of key regulators and their associated targets, future *in vitro* studies should further characterize the complex changes to posttranscriptional mechanisms contributing to mitochondrial dysfunction and driving DCM pathogenesis. Continuously expanding analytic capabilities, such as the transcriptomic and proteomic analysis of organoids used in the context of neurodevelopment and precision medicine, could be applied to DCM to further characterize pathological changes in ncRNA- and RBP-mediated posttranscriptional regulation (220, 221). While available evidence clearly highlights the crucial role of mitochondrial dysfunction in DCM and indicates the importance of ncRNA/RBP-mediated posttranscriptional regulation of gene expression in both mitochondrial dysfunction and DCM, future studies will need to bring together these insights and further define the underlying mechanisms and pathways. Identifying key posttranscriptional regulators and their targets would not only improve our understanding of the complex drivers of DCM pathogenesis but would also allow the study of new therapies which could prove effective in treating DCM and other DM-associated cardiovascular complications by modulating these complex regulatory mechanisms.

## 6. Conclusion

In summary, mitochondrial dysfunction has been shown to be a key driver of DCM, by disrupting cellular mechanisms in cardiomyocytes, including energy metabolism, Ca^2+^ homeostasis and autophagy. ncRNAs and RBPs are implicated in mitochondrial health and disease and have been shown to be significantly dysregulated in DCM. Synthesizing the evidence from studies looking at ncRNAs and RBPs in DCM and other diseases, we provide an overview of key regulators and targets, but also highlight the lack of evidence regarding the impact of ncRNAs and RBPs on mitochondrial dysfunction in the context of DCM. Future studies will need to investigate the role of individual ncRNAs and RBPs and their impact on gene expression of important cardiac proteins, such as mitochondrial enzymes and ion channels. iPSC-derived cardiomyocytes emerge as a promising *in vitro* model allowing the study of disease- and patient-specific cells of human origin. Gaining further insights into the exact posttranscriptional mechanisms driving mitochondrial dysfunction in DCM could identify promising drug targets, thereby contributing to the vital search for new therapies to improve clinical outcomes for patients suffering from DCM and other DM-associated complications.
